# The Relationships of Self-Sustained English Learning, Language Mindset, Intercultural Communicative Skills, and Positive L2 Self: A Structural Equation Modeling Mediation Analysis

**DOI:** 10.3390/bs14080659

**Published:** 2024-08-01

**Authors:** Luxi Yang, Hui Wang, Hao Zhang, Haiying Long

**Affiliations:** 1 School of Foreign Languages and Literatures, Chongqing Normal University, Chongqing 401331, China; 2 Department of Psychology, McKendree University, Lebanon, IL 62254, USA; hwang@mckendree.edu; 3 Department of Basic Education, Chongqing Industry and Trade Senior Technical School, Chongqing 401329, China; 13436087589@163.com; 4 Department of Educational Psychology, University of Kansas, Lawrence, KS 66045, USA; hlong@ku.edu

**Keywords:** learning English as a second language, self-sustained learning, intercultural communicative skills, language mindset, positive L2 self, Structural Equation Modeling, indirect effects

## Abstract

Learning English as a second language (ESL) has garnered significant attention from researchers and practitioners over the past few decades, with numerous ESL learning outcomes examined in the literature. However, self-sustained learning (SSL), a crucial factor in promoting students’ sustained learning and development within a sustainable society, has long been overlooked. To deepen the understanding of SSL, especially in the context of ESL in China, this study examined the direct and indirect effects of intercultural communicative skills, language mindset, and positive L2 self on sustained English learning among 1238 Chinese college students through descriptive statistics and a Structural Equation Modeling (SEM) mediation analysis. The results indicated that Chinese college students exhibited a strong language mindset and positive L2 self. They also scored high in intercultural communicative skills and long-term self-sustained English learning. SEM analysis showed that, after controlling for students’ demographic characteristics, both intercultural communicative skills and language mindset positively predicted positive L2 self and self-sustained English learning. Moreover, intercultural communicative skills and language mindset had significant and positive indirect effects on self-sustained English learning through positive L2 self, underscoring the significant mediating role of positive L2 self in the relationships between intercultural communicative skills, language mindset, and self-sustained English learning. These findings suggest that, to promote self-sustained learning among English learners, instructors should enhance students’ intercultural communicative skills, foster a growth language mindset, and cultivate positive language learning beliefs.

## 1. Introduction

English, recognized as one of the most popular official languages, is spoken around the world [1]. In countries where English is not the native language, it is commonly studied as a second language [2]. China, for example, has placed a high value on English learning, with approximately 400 million English learners. Most students in China begin studying English in school by the third grade. Additionally, English is a compulsory course for non-English major undergraduate students during the first two years of their studies at both 3-year and 4-year colleges, as well as for non-English major graduate students during the first year of their post-graduate studies [3,4]. 

In the past few decades, a substantial body of research has examined the outcomes of English learning as a second language (ESL) (In this article, we used learning English as a second language (ESL) and learning English as a foreign language (EFL) interchangeably), including ESL achievements [5,6,7], motivation and attitudes toward learning ESL [8], beliefs about learning ESL [6,9], self-regulated learning, self-efficacy beliefs [10], and identity construction [11]. However, one learning outcome that has long been overlooked in the literature is self-sustained learning (SSL). Originally understood from the perspective of education for sustainability, SSL is defined as “the persistent, self-initiated pursuit of expertise development in one’s subject area” (p. 2) [12]. It is considered a component of effective teaching, transforming passive student learning into an active pursuit of knowledge both inside and outside the classroom. Engaging in SSL equips students to take control of their own learning, fostering intrinsic motivation for mastering knowledge and encouraging lifelong learning [12,13,14]. 

Learning English as a second language is a long-term endeavor characterized by numerous challenges and setbacks. Consequently, it requires self-initiated efforts, patience, and persistence [15]. This is especially true in countries where the native language is significantly different from English, such as China. Despite the significance of SSL in education, very few studies have focused on this construct in the literature. Among the studies on SSL, two discussed the construct from the learning ecology framework, exploring its role in adolescents’ development of interest and technological fluency [13,14]. One study reflected on the researcher’s beliefs about excellence in second language teaching in the United States, advocating for SSL in student language learning [16]. Another proposed a framework to promote SSL in higher education [12]. Yet, no studies to date have examined SSL in the context of ESL among Chinese college students. Therefore, it remains unknown how Chinese college students engage in SSL or continue to study English outside of their classes when they are taking English classes and whether they maintain the intention to learn English when they do not enroll in English classes. Furthermore, no research has investigated the variables that predict SSL, even though there is abundant literature on factors influencing other key English learning outcomes and variables that are similar to SSL. 

The previous literature has consistently suggested that key English learning outcomes are influenced by general motivational beliefs, including the mindset about language learning, and self-concepts. These beliefs and concepts not only drive student learning and affect how students think and act but also sustain student learning, nurturing active, autonomous, and lifelong learners [8,17,18,19]. Implicit theories of intelligence suggest that students’ beliefs about their intelligence, or their mindsets, significantly impact their learning by influencing their responses to challenges and setbacks, leading to either resilient or maladaptive patterns [20,21]. Dweck (2006) identified two fundamental types of mindsets as either fixed or growth-oriented. Individuals with a growth mindset believe that skills can be acquired and enhanced through committed effort and perseverance [22]. Consequently, they focus on their capacity for change, advancing the pursuit of resilient and adaptive objectives. On the contrary, those with a fixed mindset perceive abilities as innate and unalterable, which drives individuals toward competitive and maladaptive goals [23,24]. The research has demonstrated that students with a fixed mindset are often associated with negative learning outcomes, such as fear of failure and feedback avoidance, while those with a growth mindset are linked to positive learning outcomes, such as mastery goals, higher motivation, and sustained learning [22,23,24]. Applying implicit theories of intelligence to the context of language learning, researchers have developed the construct of language mindset, which includes not only fixed or malleable language intelligence but also general language intelligence beliefs and L2 aptitude [25]. Examining the effects of these beliefs is particularly meaningful in the educational context of China, where Confucian philosophy, which advocates for efforts or the growth mindset in learning, predominates [26,27]. 

Furthermore, Brown (2004) defined self-concept as a “process of thinking about one’s own experiences and behaviors, then contemplating one’s thought processes, and the need for self-acceptance and ego protection” (p. 123) [20]. In other words, self-concepts reflect the perceptions and beliefs that individuals hold about themselves [28]. More recently, scholars have suggested that students’ academic self-concept might vary across different subjects [29]. Accordingly, in the context of ESL, Lake (2015) proposed a construct of positive L2 self based on positive psychology that focuses on people’s optimal functioning [30]. The construct includes three subdimensions: interest in the L2 self, which refers to the tendency to perceive the learning of a second language as fascinating and enjoyable, harmonious passion for L2 learning, which indicates a strong inclination toward activities related to language that are favored or loved, and the mastery of L2 goal orientation, which refers to an individual’s purpose that focuses on achieving substantial progress in learning [30]. Given the emphasis on the interdependence of individuals over individualism in many Asian cultures, it is worthwhile to explore how L2 self-concepts affect language learning outcomes in those cultural contexts. 

English serves as a means of communication between different cultures, a function that has become especially important in the current globalized world [1,2]. Considering the close relationship between language and culture, learning a second language involves learning the target culture and how to communicate within that culture [31,32]. A person’s competence or skill of communicating across different cultures significantly affects many language learning outcomes, such as willingness to communicate and persistence in language learning [33,34]. Individuals with a high level of intercultural communicative skills are more willing to engage in intercultural interactions and become increasingly confident in their L2 learning, which, in turn, can motivate them to persist longer [33,34]. Intercultural communicative skills are particularly important for Chinese L2 learners for two reasons: first, L2 classes in China often focus more on English reading and writing than on listening and speaking, resulting in a generation of English learners with a low level of intercultural communicative skills; second, communication between China and other countries via English has significantly increased in the past few decades, making it essential to understanding the role of intercultural communicative skills in L2 learning outcomes [35,36]. 

Previous literature on learning outcome constructs that are similar to SSL, such as self-regulated English learning and persistence in English learning, were found to have significant relationships with the variables examined above [6,37,38]. In the same vein, we hypothesized that SSL is significantly predicted by these variables. The current study seeks to investigate these relationships through a quantitative study, which includes descriptive statistics and a Structural Equation Modeling (SEM) analysis. The findings of this study will illuminate college students’ English learning not only in the Chinese context but also in other ESL contexts.

## 2. Literature Review

### 2.1. Self-Sustained Learning

Over two decades ago, UNESCO (2002) called for education for sustainability by stating “Improving the quality and coverage of education and reorienting its goals to recognize the importance of sustainable development must be among society’s highest priorities” (p. 9) [39]. This not only heightened the awareness of researchers and educators about keeping our world sustainable through education but also posed a critical question to schools and teachers about how to make education itself sustainable to support a sustainable society [40,41,42]. Researchers have argued that the current educational paradigm is not oriented toward equipping students with the competencies needed to address the challenges of a sustainable world that is becoming more complex and interdependent [41,42]. Consequently, educators need to adopt a transformative learning paradigm that emphasizes deep and “critically reflective” learning (p. 9) and sustainable education that is “sustaining, tenable, healthy, and durable” (p. 2) [41]. Rather than focusing on testing and competition, this new paradigm underscores the importance of community, engagement, real purpose, participation, ownership, democracy, openness, and environment, integrating all these aspects to ensure student success in education [42,43].

Under the context of education for sustainability, the concept of self-sustained learning (SSL) has been identified as a key high-level learning outcome [12]. It was further discussed by Barron (2006) in her study on adolescents’ development of technological fluency within and outside of school contexts [13]. Grounded in sociocultural and activity theory [44,45], SSL focuses on the key role that individuals play in their development, for instance, their temperament and personality shape how people respond to them. It also aligns with theories of identity development [46] and interest development [47]. According to identity development theories, learning that transforms identity is different from everyday learning by making individuals perceive the possibility of becoming someone new. Similarly, interest develops from a self-initiated learning process that seeks opportunities for new activities, knowledge, and relationships. With these theoretical perspectives, SSL describes a type of learning that is directly derived from the self-initiatives of the learners, showing their intrinsic interest in a subject area and motivating them to seek knowledge and expertise independently and persistently beyond traditional learning environments [12,13,14]. 

Self-sustained learning is recognized as an effective learning approach that facilitates students’ active learning and fosters genuine learning. It enables students to acquire, understand, retain, and apply knowledge on their own, leading to the mastery of concepts and the development of core competencies, including problem-solving ability, creativity, and critical thinking [48,49,50]. By learning in a self-sustained manner, students, often perceived as passive learners in the classroom, are empowered to control their learning, think independently, and actively interact with their peers. They are also more inclined to extend in-class learning time to outside spaces, using both formal and informal learning opportunities to acquire knowledge and skills [13,49,51].

Self-sustained learning shares many similarities with other learning outcomes, such as self-regulated learning, persistence, grit, and lifelong learning. Both SSL and self-regulated learning emphasize the key role of “self” or the active role of individuals in the learning process. Persistence and self-sustained learning stress continual effort and perseverance, while grit also includes passion and interest in long-term goals [52]. In contrast, both SSL and lifelong learning highlight the importance of an extended time commitment in the learning process [9]. However, compared to research on its similar outcomes, SSL has received much less attention, with only a few studies examining it [12,53]. For instance, Barron (2007) designed a computer science curriculum based on a new school–university partnership as an intervention to enrich students’ learning ecologies and inspire their SSL [14]. Yang (2015) analyzed possible issues teachers usually encounter in nurturing students’ SSL in the classroom and suggested three strategies to address these issues, including inquiry-based scaffolding tasks, engaging classroom dialogues, and engaged critical reflections [12]. For instance, tasks can be implemented through problem-based learning, project-based learning, or case studies, aiming to facilitate deeper learning, enhance the retention of knowledge, and improve lifelong learning skills. Checketts (2019) reflected on his teaching philosophy in teaching English as a second language and reviewed the literature on teaching communication strategies and the pragmatics of greetings in second language classes, aiming to foster students’ SSL [16]. 

Due to the dearth of research on SSL, to understand this variable and its relationships with other variables in the context of ESL, researchers must refer to the ESL literature on variables that are similar to SSL. For instance, very recently, Bai and Wang (2023) examined self-regulated learning with 690 4th grade ESL learners in Hong Kong and found that self-regulated learning in ESL had a significant relationship with growth mindset. The effect of growth mindset on self-regulated learning was stronger than that of other variables, such as self-efficacy and intrinsic value [6]. Khajavy et al. (2020) studied language mindset and grit among over 1000 college ESL learners and concluded that a growth mindset positively predicted one component of grit—perseverance of effort—while a fixed mindset negatively predicted the other component of grit—consistency of interest [37]. Additionally, studied with 436 Chinese ESL learners, Wang (2023) found that language mindset significantly predicted positive L2 self, and both variables significantly predicted academic resilience in EFL learning [38]. 

### 2.2. Intercultural Communicative Skills

Intercultural communicative skills are a key component of intercultural competence (ICC), which is regarded as an essential skill in higher education [54,55]. The construct of ICC has been studied in different cultures [56,57], including China, where scholars have conceptualized, defined, and developed measures of ICC specific to the Chinese context [58,59,60,61]. Wu et al. (2013), for instance, defined ICC from six aspects: knowledge of self, knowledge of others, attitudes, intercultural communicative skills, intercultural cognitive skills, and awareness [62]. Among these, intercultural communicative skills that focus on the skills individuals use to communicate across different cultures are deemed the most critical aspect of ICC, because the concept of ICC originated from the notion of communicative competence [57]. These skills are considered one of the ultimate goals of students’ English learning [63,64] and a significant component of the language proficiency of students who learn English as a second language [65,66].

Past research has demonstrated that intercultural communicative skills positively impact language learning motivation, willingness to communicate, and language competence of ESL learners [67,68,69]. For instance, Tran and Duong (2018) implemented a 13-week intercultural language communicative teaching model with ESL learners in Vietnam and reported significant improvements in the learners’ language and intercultural competence [69]. Badrkoohhi (2018) found a negative relationship between intercultural communicative skills and demotivation among 60 Iranian EFL learners, suggesting that intercultural communicative skills functioned as a protective factor against the demotivation of EFL learners [70]. Additionally, Kanat-Mutluoglu (2016) observed a moderate association between intercultural communicative competence and the ideal L2 self among 173 college students in Turkey [71]. Although these studies provided valuable insights into the topic, they were constrained by a limited sample size and a narrow range of examined variables, particularly the lack of sustained language learning outcomes, such as SSL. 

### 2.3. Language Mindset

According to the notion of language mindset proposed by Lou and Noels (2016), it consists of three key components: general language intelligence beliefs that focus on whether language intelligence is considered fixed or malleable, second language aptitude that refers to whether the ability to learn a language is viewed as changeable through effort or as an innate, fixed trait, and beliefs related to age sensitivity and language learning that address whether the ability to learn a language can be cultivated up to a certain age and remained fixed thereafter [72]. 

Studies have indicated that language mindset plays a crucial role in language learning. Specifically, a growth language mindset is positively related to language learners’ learning goals, engagement, and achievements [25,72,73,74]. For example, Lou and Noels (2016) demonstrated that learners with a growth language mindset endorsed learning goals more strongly, regardless of their perceived language competence. This mindset resulted in a more persistent intention to continue learning the language [72]. In a subsequent study, the same researchers (2017) investigated the relationships of language mindset, intercultural interactions, and cultural adaptation. They found that students with a fixed mindset experienced a higher level of anxiety about the target language community and had poorer intercultural interactions and cross-cultural adaptation. In contrast, students with a growth mindset exhibited lower anxiety, better intercultural interactions, and more successful cultural adaptation [73].

In addition, multiple studies have suggested that students with a growth mindset are more likely to have positive self-concepts, including a positive L2 self, in subjects such as math [74,75]. However, these studies have yielded mixed conclusions in the context of language learning. Some research has indicated a positive connection between language learners’ growth mindset and their self-concept [74], while other studies have argued that students’ mindsets are not significantly correlated with self-concept [74]. Therefore, additional research is required to examine whether students’ language mindsets can influence their self-concept, which, in turn, could predict their SSL. 

### 2.4. Positive L2 Self

Multiple studies have found that positive L2 self is positively related to various aspects of English learning, including academic resilience in English learning, L2 self-efficacy, and L2 proficiency [30,38]. For example, Lake (2015) investigated the relationships between positive L2 self, L2 self-efficacy, and L2 proficiency among 539 first- and second-year Japanese college students in a two-year private college and a four-year private university in Western Japan [30]. The results indicated that positive L2 self significantly predicted L2 self-efficacy, which, in turn, influenced L2 proficiency. This line of research aligns with studies on the ideal L2 self, a core component of the L2 Motivational Self System [76]. A meta-analysis of 32 research publications concluded that the ideal L2 self is a significant and strong predictor of subjective intended effort [77]. Furthermore, a recent study involving 198 Tibetan high school ESL learners supported the same findings, suggesting a stronger effect of the ideal L2 self on learning effort than on L2 achievement [78]. Fathi et al. (2023) investigated the effects of foreign language enjoyment, ideal L2 self, and ICC on L2 willingness to communicate among 601 EFL learners in Iran and found that all the variables of interest (i.e., FLE, ideal L2 self, and ICC) directly predicted L2 willingness to communicate [67].

The mediating role of the L2 self, including both the positive L2 self and the ideal L2 self, has been consistently demonstrated in the literature. For instance, Wang (2023) found that the positive L2 self significantly mediated the relationships between the EFL classroom social climate and academic resilience in EFL learning [38]. Within the theoretical framework of the L2 Motivational Self System [76], Ebn-Abbasi et al. (2024) observed a significant mediating role of the ideal L2 self between L2 grit and willingness to communicate [79]. Similarly, Sadoughi et al. (2023) further indicated a significant mediating effect of the ideal L2 self between a growth mindset and academic engagement among 384 EFL learners in Iran [80]. While these studies significantly contribute to our understanding of the L2 self in general, they primarily focused on outcome variables other than sustained learning outcomes, such as SSL. Additionally, although research on the ideal L2 self sheds light on the concept of positive L2 self, these two concepts are distinct. Therefore, more research is needed to achieve a comprehensive understanding of the mediating role of the ideal L2 self in ESL learning.

## 3. The Current Study

**Figure 1:**
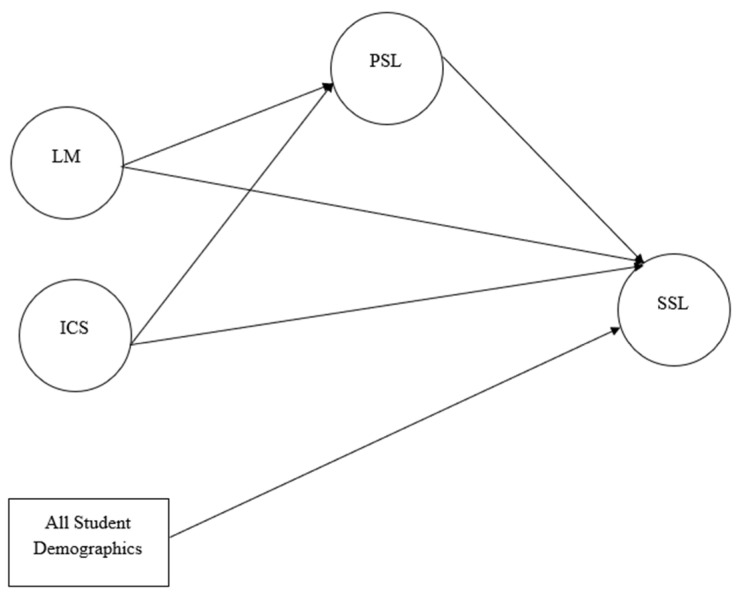
Structural Equation Model. LM: language mindset, ICS: intercultural communicative skills, PSL: positive L2 self, and SSL: self-sustained learning.

Based on the above literature review, we created a statistical model (see Figure 1) about the relationships of the variables of interest, aiming to understand the direct and indirect relationships among these variables. In the model, language mindset (LM) and intercultural competence (ICC) are two exogenous variables, positive L2 self is the mediator, and SSL is the endogenous variable. We seek to test the direct effects of LM and ICC on positive L2 self (RQ2); the direct effects of LM, ICC, and positive L2 self on SSL (RQ3); and the indirect effects of LM, ICC, and SSL through the mediating effect of the positive L2 self (RQ4). All student demographic variables, such as their English and non-English major and English proficiency, were included in the model as control variables (see Section 4.2.5; students’ demographic variables were not listed in Figure 1 due to limited space. Please refer to all students’ demographic variables in Table 1). Additionally, we used descriptive statistics to understand Chinese ESL learners’ SSL, LM, ICC, and positive L2 self (RQ1). Overall, we addressed the following research questions in the current study: 

RQ1. Do Chinese ESL learners engage in self-sustained English learning? What type of language mindset do they possess? What are their levels of positive L2 self and intercultural communicative skills? 

RQ2. Do language mindset and intercultural communicative skills of Chinese ESL learners significantly predict their positive L2 self?

RQ3. Do language mindset, intercultural communicative skills, and positive L2 self of Chinese ESL learners directly predict their self-sustained English learning?

RQ4. Does language mindset and intercultural communicative skills of Chinese ESL learners indirectly predict their self-sustained English learning through positive L2 self?

## 4. Methods

### 4.1. Participants

Participants (*N* = 1238) were mainly recruited through the assistance of their English teachers from four universities in Chongqing, China. Students who were interested in the study self-selected to participate. Therefore, the sample was limited by self-selection bias and was constrained to students in one area in China. However, the sample’s representativeness was enhanced by including four different types of universities: a flagship comprehensive university, a normal or teacher university, a science and technology university, and a private university. Most of the participants (68.7%) were female, undergraduate students (65.3%), non-English majors (75.9%), and had studied English as a second language for 5–10 years (61.1%, see Table 1). 

### 4.2. Measures

#### 4.2.1. Language Mindset

Language mindset was measured by Wang et al.’s (2021) Chinese version of the Language Mindsets Inventory, which was originally developed by Lou and Noels (2017) and has been popularly adapted in studies focusing on English language learning. Previous studies have shown good reliability and validity of this instrument in different languages [25,37,81], including the Chinese version [82]. The instrument consists of 9 items, with 3 items designed to measure each of the three dimensions—second language aptitude beliefs, age sensitivity beliefs about language learning, and general language intelligence beliefs. Example items include “You can always change your foreign language ability”; “In learning a foreign language, if you work hard at it, you will always get better”; “How good you are at using a foreign language will always improve if you really work at it”; and “Everyone can do well in foreign language if they try hard, whether they are young or old”. All items were based on a 5-point Likert scale (1—Strongly Disagree; 5—Strongly Agree). Cronbach’s alpha was 0.94 for all 9 items and 0.83, 0.86, and 0.88 for the items measuring the three dimensions in this study. 

#### 4.2.2. Intercultural Communicative Skills

Intercultural communicative skills were measured by a section on the same construct in the Assessment of Intercultural Competence of Chinese College Students (AIC-CCS), which was developed by Wu et al. (2013) for Chinese college students in a Chinese context and has been widely used in China [83,84,85]. Previous studies have shown that the instrument has good reliability and validity. A total of 9 items measures intercultural communicative skills. Participants are asked to rate themselves about their skills based on a 5-point Likert scale (1—Very Low; 5—Very High). Example items include “the skill of consulting with foreigners when misunderstandings occur”; “the skill of treating foreigners politely”; “the skill of negotiating with others”; and “the skills of communicating using body language or any other non-linguistic methods”. Cronbach’s alpha of the 9 items in this study was 0.94. 

#### 4.2.3. Positive L2 Self

Positive L2 self was assessed by Wang’s (2023) Chinese version of Lake’s (2015) measure, which has 21 items [30,38]. Seven items are designed to measure one of the three dimensions: interest in L2 self, harmonious passion for L2 learning, and mastery of L2 goal orientation [38]. Previous studies have shown good reliability and validity of this instrument. All items were based on a 5-point Likert scale (1—Definitely Not True Of Me; 5—Definitely True Of Me). Example items include “English lessons are enjoyable”; “I am passionate about learning English”; “My goal is to learn as much as possible in this class”; and “I like to study new topics in this class”. Cronbach’s alpha was 0.98 for all 21 items and 0.96, 0.95, and 0.95 for the items measuring the three dimensions in this study.

#### 4.2.4. Self-Sustained English Learning

The self-sustained English learning scale was developed by the authors of this study based on the literature review on SSL [12,13,14,16]. The scale consists of two components: short-term self-sustained English learning and long-term self-sustained English learning. The short-term component includes 7 items related to students’ SSL when they are taking English classes. Example items include “I keep learning English after class”; “I practice English with my peers during my after-class time”; “I learn more about English and American cultures during my after-class time”; and “I read English materials (e.g., English magazines, news, and novels) during my after-class time”. The long-term component consists of 4 items related to students’ self-sustained English learning after completing their English classes or after graduation. Example items include “I will continue to learn English in my future jobs”; “I will continue to learn English after I graduate from college”; “I will continue to learn English after I complete all my English classes”; and “I plan to learn English for the rest of my life”. All items were based on a 5-point Likert scale (1—Strongly Disagree; 5—Strongly Agree). This instrument underwent a few procedures to ensure its reliability and validity before being used in this study, such as expert reviews to provide validity evidence based on content, cognitive interviews to provide validity evidence based on response processes, and Exploratory Factor Analysis (EFA) to provide validity evidence based on the internal structure. For instance, in the original development and validation study with 300 Chinese college students who learned English as a second language, the EFA results confirmed the two factors in the original conceptualization of this measure, and Cronbach’s alpha of the 11 items was 0.89. In this study, Cronbach’s alpha was 0.94, 0.90, and 0.89 for all 11 items and items measuring the two components, respectively.

#### 4.2.5. Demographic Background

Students’ demographic backgrounds were measured by indicating their sex; grade; school; their self-evaluation of English proficiency level; the time spent learning English; the frequency of being in contact with English native speakers; and whether they were undergraduate students, English majors, or had been abroad. 

### 4.3. Procedure

The study was approved by the Institutional Research Board at the first author’s institution and complied with the Declaration of Helsinki. The survey that included all the measures and the demographic questions was administered at www.wjx.cn, the most widely used secure online survey platform in China. Participants first read the information statement about the study, signed the informed consent, and agreed to participate in the study. They then completed all the measures and the demographic questions. No identifying information was collected. 

### 4.4. Data Analysis

**Figure 2:**
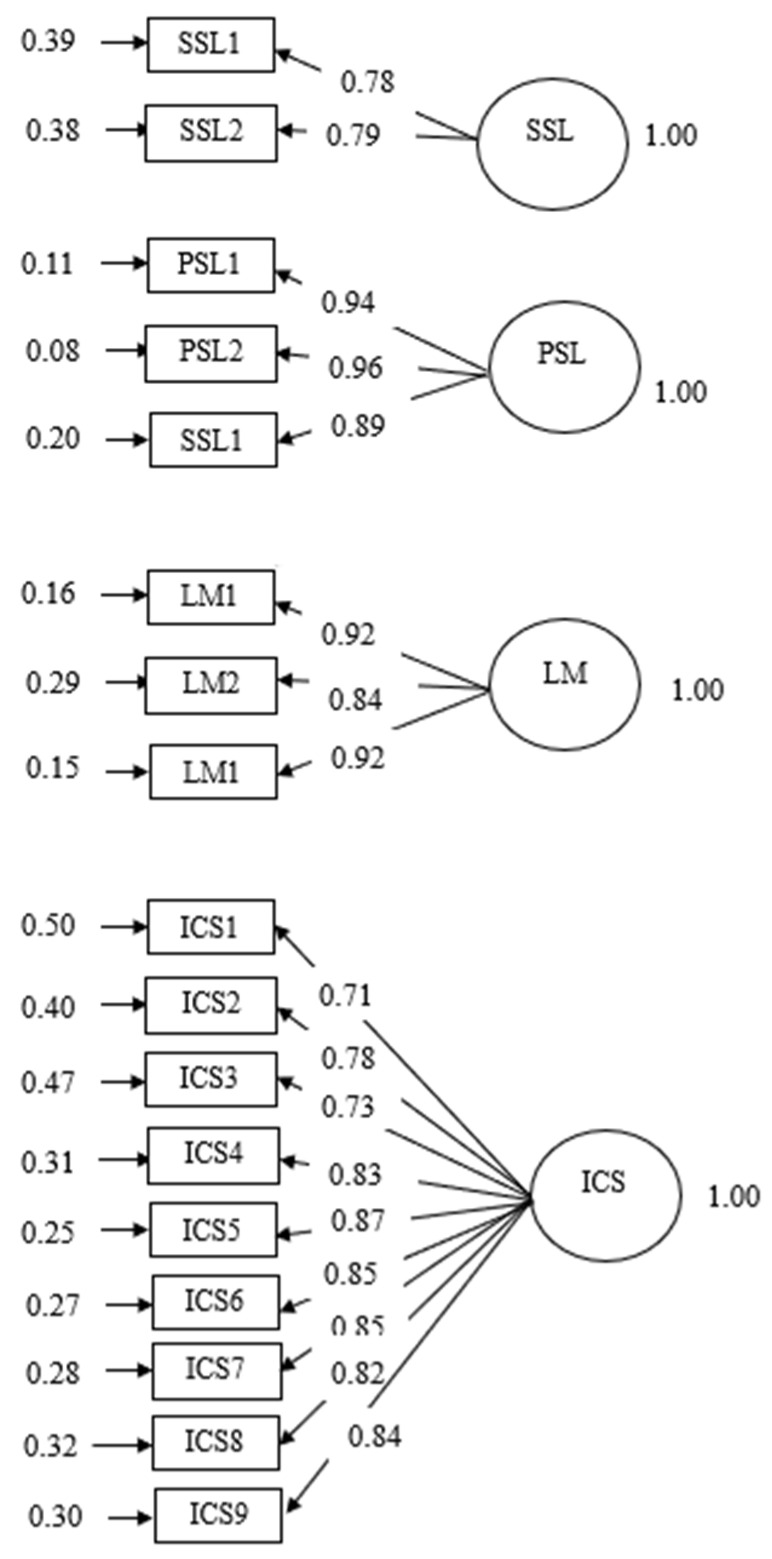
Measurement model. LM: language mindset, ICS: intercultural communicative skills, PSL: positive L2 self and SSL: self-sustained learning.

Before any analysis was conducted, the data were checked for normality and outliers, and the normality assumption was found not to be violated. The main analysis method we used was latent variable Structural Equation Modeling (SEM) mediation analysis, which can examine both measurement and structural models simultaneously [86,87] and provide an unbiased estimate of direct and indirect effects [88]. Two steps were carried out based on SEM researchers’ recommendations [88,89]: the first step was a Confirmatory Factor Analysis (CFA) of all the latent variables, which aimed to ensure sufficient construct validity of the measurement model (see Figure 2); the second step was SEM, which aimed to understand the structural relationships among the four latent variables, including the mediating effects of positive L2 self (see Figure 1). In this SEM model, language mindset and intercultural communicative skills were treated as exogenous variables, positive L2 self was the mediator, and self-sustained English learning was the endogenous variable. Students’ demographic information was used as control variables, which included sex, degree, major, self-rated English proficiency level, time spent English learning, going abroad, or contact with foreigners. 

The main analyses of CFA and SEM were performed in Mplus 8.10 [90]. Parceling was employed in the analyses to create parceled items as indicators of three latent constructs: language mindset, positive L2 self, and self-sustained English learning. More specifically, parcels were created as the average of items that measure each dimension of the construct. For instance, two parcels were formed for self-sustained English learning that corresponds to short-term and long-term self-sustained English learning. Despite a few weaknesses of parceling, this approach has many advantages, such as parceled items leading to simpler models and a better fit [91,92]. In interpreting the results of CFA and SEM, we used model fit indices such as the Comparative Fit Index (CFI), Tucker–Lewis Index (TLI), Root Mean Square Error of Approximation (RMSEA), and Standardized Root Mean Square Residual (SRMR). A model is often considered excellent if the CFI and TLI are 0.95 or above, RMSEA is 0.06 or below, and SRMR is 0.08 or below [93,94]. The STDYX function in Mplus was used to obtain all standardized coefficients. The bootstrap Confidence Interval method was used to test indirect effects, with 5000 bootstraps, because it can control Type I errors while yielding an accurate value [86,95]. 

## 5. Results

### 5.1. Descriptive Statistics and Correlations

Descriptive statistics of the parceled items of language mindset, positive L2 self, and self-sustained English learning and all nine items of intercultural communicative skills are reported in Table 2. Overall, students had a strong language mindset, with a slightly higher level of beliefs related to age sensitivity when learning English than their beliefs related to second language and general language intelligence. Students’ scores in the three components of positive L2 self were strong, suggesting very positive perceptions related to their interest in L2 self, harmonious passion for L2 learning, and mastery of L2 goal orientation. Additionally, students scored higher in long-term self-sustained English learning than in short-term self-sustained English learning, supporting their stronger intention to continue to learn English even after they stop taking English classes or after graduation. Among the nine items that measure intercultural communicative skills, students rated the highest as their ability to politely treat foreigners but rated the lowest as their ability to negotiate with others. Correlations of the four latent variables were also calculated (see Table 3). The results indicated that all variables were significantly correlated, and the correlations ranged from 0.34 to 0.50, showing small to moderate correlational relationships among the latent variables.

### 5.2. CFA

The CFA model with four latent variables showed a good fit: CFI: 0.90, TLI: 0.88, RMSEA: 0.12, 95% CI [0.118, 0.127], and SRMR: 0.05. To account for the correlations of a few indicators in the intercultural communicative skills measure, we added their correlated residuals in the model, such as ICC1 and ICC2 and ICC1 and ICC3. This significantly increased the model fit: CFI: 0.95, TLI: 0.94, RMSEA: 0.087, 95% CI [0.082, 0.092], and SRMR: 0.05. All factor loadings were significant and strong, ranging from 0.71 to 0.96 (see Figure 2). All these suggested a good CFA model, which allowed us to subsequently run the SEM mediation analysis with confidence.

### 5.3. SEM

**Figure 3:**
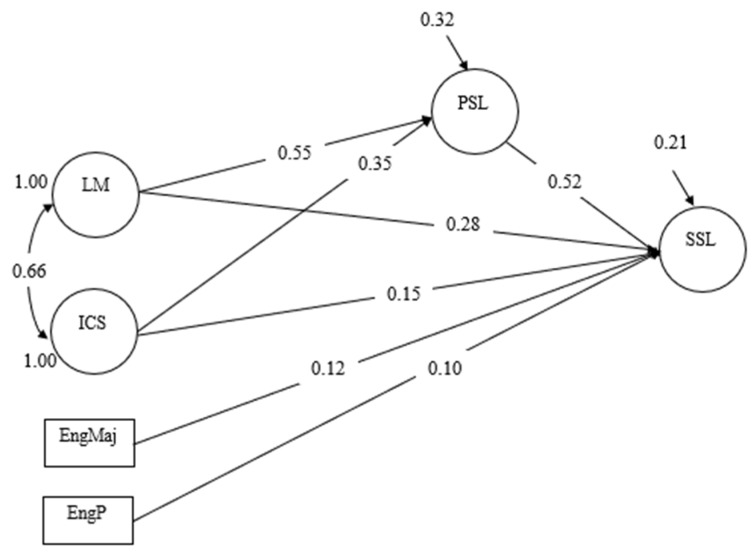
SEM results. LM: language mindset, ICS: intercultural communicative skills, PSL: positive L2 self, SSL: self-sustained learning, EngMaj: English major and EngP: English proficiency level.

Language mindset and intercultural communicative skills were both significant and positive predictors of positive L2 self, suggesting that students with higher levels of language mindset and better intercultural communicative skills tended to exhibit a more positive L2 self. The former variable (β = 0.55, *p* < 0.001) had a stronger effect than the latter one (β = 0.35, *p* < 0.001), suggesting that language mindset was a better predictor of positive L2 self than intercultural communicative skills. These two variables explained a large portion (68.1%) of the variance in positive L2 self, supporting the robustness of the statistical model. Furthermore, language mindset, intercultural communicative skills, and positive L2 self all had significant direct effects on self-sustained English learning. Among these three variables, positive L2 self had the strongest effect (β = 0.52, *p* < 0.001), followed by language mindset (β = 0.28, *p* < 0.001) and intercultural communication skills (β = 0.15, *p* = 0.001), demonstrating that positive L2 self was the best predictor of self-sustained English learning. Additionally, language mindset and intercultural communication skills had significant indirect effects on self-sustained English learning through positive L2 self (see Figure 3). The indirect effects of language mindset and intercultural communicative skills were both very strong, with the former indirect effect being 0.29 (*p* < 0.001, 95% CI [0.21, 0.36]), which was stronger than that of intercultural communication skills (β = 0.19, *p* < 0.001, 95% CI [0.15, 0.25]). These findings suggest that language mindset and intercultural communicative skills had significant impacts on positive L2 self, which, in turn, affected self-sustained English learning. Of the students’ demographic characteristics treated as control variables, only students’ status as an English major (β = 0.12, *p* < 0.001) and their self-rated English proficiency level (β = 0.10, *p* = 0.001) were significant predictors of self-sustained English learning, with both exhibiting small and comparable effects (other non-significant demographic characteristics were not included in Figure 3 to save space). This model, incorporating both direct and indirect effects, as well as the effects of the two control variables, explained a substantial portion (79.2%) of the variance in self-sustained English learning, corroborating a very strong structural model. 

## 6. Discussion

This study is the first empirical study to examine self-sustained learning and its predictors among Chinese college students who study English as a second language. The findings indicated that students scored higher in long-term self-sustained English learning compared to short-term self-sustained English learning. This suggests a strong intention to continue learning English beyond their compulsory English courses and after graduation. This trend may be attributed to the high value placed on English proficiency in Chinese society, where many companies and government units require employees to be proficient in English. Additionally, given that many college students in China view English as a practical tool for various aspects of life, such as work and travel [8], it is not surprising to find that they wish to maintain their English after completing their English classes and even after graduation.

Furthermore, this study showed that Chinese students had an overall strong language mindset and a positive L2 self, which is consistent with previous findings. For instance, Liu (2007) found that the majority of college students in China had moderately or strongly positive attitudes toward English learning [8]. We also found that students had slightly higher beliefs related to age sensitivity compared to their beliefs about second language aptitude and general language intelligence. Because the three items measuring age sensitivity beliefs in general focus on the importance of efforts over age (e.g., “Everyone can do well in foreign language if they try hard, whether they are young or old”), a higher score in this measure suggests that students prioritize the importance of effort in learning English over the belief that their English learning is limited by age. This is in line with the Chinese culture, which espouses the significance of effort in learning [96]. This study also indicated that Chinese college students felt most confident in their ability to treat foreigners politely but felt least confident in their ability to negotiate with others. Politeness in China is a highly valued quality, and being polite in English can often be easily achieved by using phrases like “please” or “thank you”. In contrast, negotiating with others is inherently more challenging than simply conversing or being polite, as it requires not only language competencies but also strategies to persuade or convince others.

This study demonstrated that both intercultural communicative skills and language mindset significantly predicted a positive L2 self, which, in turn, significantly predicted self-sustained English learning. These findings are consistent with previous research indicating that language mindset, intercultural communicative skills, and positive L2 self were significant positive predictors of various L2 learning outcomes, including the intention to continue to learn English and L2 proficiency [25,30,72,73]. Interestingly, the study found that language mindset was a stronger predictor of a positive L2 self than intercultural communicative skills, while a positive L2 self was a stronger predictor of self-sustained English learning than language mindset. This suggests that students’ beliefs about language play a more critical role than their communication skills in shaping positive attitudes toward learning English as a second language. Conversely, students’ positive attitudes toward learning English and their self-perceptions are more crucial than their beliefs about language in promoting short-term and long-term self-sustained learning in English. These findings imply that teachers should focus not only on fostering students’ positive attitudes toward learning English and their beliefs about English but also on training students’ intercultural communicative skills. This balanced approach can help enhance both their positive L2 self and their self-sustained learning.

This study further found that students’ self-rated English proficiency level and being an English major significantly predicted self-sustained English learning. Previous studies treated English proficiency level as a dependent variable and showed that language mindset, positive L2 self, and intercultural communicative skills were predictors of English proficiency level [30,38,69,97]. This study suggests that the relationship between English proficiency level and other L2 learning outcomes may be bidirectional. Additionally, it is not surprising to find that being an English major was a significant predictor of self-sustained English learning. English majors not only spend more time and effort studying English while taking classes but also use English more frequently after graduation, primarily in their future jobs. This increased exposure and practice contributed to their higher levels of self-sustained English learning compared to non-English majors. 

The most interesting finding of this study was that both intercultural communicative skills and language mindset had significant indirect effects on self-sustained English learning through positive L2 self. This suggests that, while improving students’ language mindset and intercultural communicative skills can enhance their self-sustained English learning, cultivating a positive L2 self may be a more effective strategy. The significant role of positive L2 self in learning ESL has been consistently reported in previous studies [30,38]. The positive L2 self comprises three components: interest in L2 self, harmonious passion for L2 learning, and mastery of L2 goal orientation. Teachers can focus on these aspects to nurture students’ positive L2 self in English classes. 

### Limitations 

Despite the interesting findings, this study has several limitations. First, the sample was a convenience sample, and most of the colleges sampled were from the same area. Although the type and size of these colleges and the characteristics of the students were considered in the sampling, the sample used in this study was not representative of college students in China, which limits the generalizability of the findings in this study to other contexts. Future studies should use a more representative sample in China and other countries where students learn English as a second language to determine if the same results are replicated. Second, this study used a cross-sectional design. We did not intend to make any causality claims about the relationships examined in this study; therefore, any such claims should be approached with caution or avoided. Future studies can use longitudinal designs to examine the cause-and-effect relationships among the examined variables. Third, although we collected data from both English and non-English majors, we did not analyze the data in separate samples. Future studies can use multiple-group SEM to examine the relationships among the same variables in samples of English majors and non-English majors. 

## 7. Conclusions

This study is the first in the literature to examine self-sustained learning and its predictors among Chinese college students in the context of learning English as a second language. The findings showed that students scored higher in long-term self-sustained English learning than in short-term self-sustained English learning. Additionally, students exhibited a strong language mindset, positive L2 self, and intercultural communicative skills. The study also demonstrated that intercultural communicative skills and language mindset were significant positive predictors of both positive L2 self and self-sustained English learning. Positive L2 self also significantly predicted self-sustained English learning. Furthermore, language mindset and intercultural communicative skills had significant indirect effects on self-sustained English learning through the effect of positive L2 self. These findings have significant implications for nurturing students’ self-sustained English learning and provide valuable insights into understanding intercultural competence, language beliefs, positive attitudes, and their relationships with self-sustained learning among students who learn English as a second language.

## Figures and Tables

**Table 1:** Participant demographics.

Variables	Categories	*N*	%
Gender	Female	850	68.7
	Male	388	31.3
Grade	Undergraduate	808	65.3
	Graduate	430	34.7
Major	English Major	940	75.9
	Non-English Major	298	24.1
Length of English Learning	Less than 5 years	89	7.2
	5–10 Years	757	61.1
	11–15 Year	314	25.4
	More than 15 Years	78	6.3
Self-Rated English Proficiency Level	Very Poor	43	3.5
	Poor	211	17.0
	Fair	815	65.8
	Good	37	3.0
	Very Good	132	10.7
Going Abroad	No	1149	92.8
	Yes	89	7.2
Contact with Native Speakers	No	704	56.9
	Once or more per year	286	23.1
	Once or more per month	66	5.3
	Once or more per week	149	12.0
	Once or more per day	33	2.7

*Note. N* = 1238.

**Table 2:** Descriptive statistics of all the variables.

Variables	Mean	SD
LM Parcel 1—Second Language Beliefs	3.75	0.79
LM Parcel 2—Age Sensitivity Beliefs	3.88	0.85
LM Parcel 3—General Language Beliefs	3.69	0.83
PLS Parcel 1—Interest	3.71	0.83
PLS Parcel 2—Harmonious Passion	3.66	0.83
PLS Parcel 3—Mastery L2 Goal	3.72	0.79
SSL—Short-Term	3.07	0.95
SSL—Long-Term	3.63	0.99
ICS 1	3.21	1.00
ICS2	3.55	0.88
ICS3	3.23	0.98
ICS4	3.79	0.91
ICS5	3.71	0.90
ICS6	3.71	0.89
ICS7	3.72	0.89
ICS8	3.48	0.90
ICS9	3.56	0.88

*Notes.* LM = language mindset, PLS = positive L2 self, SSL = self-sustained English learning, and ICS = intercultural communicative skills.

**Table 3:** Correlations of the latent variables.

Variables	LM	ICS	PLS	SSL
LM	-			
ICS	0.34 ***	-	-	
PLS	0.44 ***	0.40 ***	-	-
SSL	0.43 ***	0.39 ***	0.50 ***	-

*Notes.* LM = language mindset, PLS = positive L2 self, SSL = self-sustained English learning, and ICS = intercultural communicative skills. *** *p* < 0.001.

## Data Availability

Data is unavailable due to privacy or ethical restrictions.

## References

[1] Crystal D. (2003). English as a Global Language.

[2] Jenkins J. (2014). Global Englishes: A Resource Book for Students.

[3] Wei R., Su J. (2012). The statistics of English in China: An analysis of the best available data from government sources. Engl. Today.

[4] Hu G. (2005). English language education in China: Policies, progress, and problems. Lang. Policy.

[5] Bai B. (2018). Understanding primary school students’ use of self-regulated writing strategies through think-aloud protocols. System.

[6] Bai B., Wang J. (2023). The role of growth mindset, self-efficacy and intrinsic value in self-regulated learning and English language learning achievements. Lang. Teach. Res..

[7] Muñoz C. (2017). Tracing trajectories of young learners: Ten years of school English learning. Annu. Rev. Appl. Linguist..

[8] Liu M. (2007). Chinese students’ motivation to learn English at the tertiary level. Asian EFL J..

[9] Su D. (1995). A Study of English Learning Strategies and Styles of Chinese University Students in Relation to Their Cultural Beliefs and Beliefs about Learning English.

[10] Wang C., Hu J., Zhang G., Chang Y., Xu Y. (2012). Chinese college students’ self regulated learning strategies and self-efficacy beliefs in learning English as a foreign language. J. Educ. Res..

[11] Pan H., Liu C., Fang F., Elyas T. (2021). “How is my English?”: Chinese university students’ attitudes toward China English and their identity construction. Sage Open.

[12] Yang M. (2015). Promoting self-sustained learning in higher education: The ISEE framework. Teach. High. Educ. J..

[13] Barron B. (2006). Interest and self-sustained learning as catalysts of development: A learning ecology perspective. Hum. Dev..

[14] Barron B., Martin C.K., Roberts E. (2007). Sparking self-sustained learning: Report on a design experiment to build technological fluency and bridge divides. Int. J. Technol. Des. Educ..

[15] Belnap R., Bown J., Dewey D., Belnap L., Steffen P., MacIntyre P., Gregersen T., Mercer S. (2016). 12 project perseverance: Helping students become self-regulating learners. Positive Psychology in SLA.

[16] Checketts H.B. (2019). Guiding Language Students to Self-Sustained Learning. Unpublished Thesis.

[17] Brown H.D. (2000). Teaching by Principles: An Interactive Approach to Language Pedagogy.

[18] Eccles J.S., Wigfield A. (2002). Motivational beliefs, values, and goals. Annu. Rev. Psychol..

[19] Wigfield A., Eccles J.S., Fredricks J.A., Simpkins S., Roeser R.W., Schiefele U., Lerner R. (2015). Development of achievement motivation and engagement. Handbook of Child Psychology and Developmental Science.

[20] Brown E. (2004). The relationship of self-concepts to changes in cultural diversity awareness: Implications for urban teacher educators. Urban. Rev..

[21] Zarrinabadi N., Rezazadeh M., Karimi M., Lou N.M. (2022). Why do growth mindsets make you feel better about learning and yourselves? The mediating role of adaptability. Innov. Lang. Learn. Teach..

[22] Dweck C.S. (2006). Mindset: The New Psychology of Success.

[23] Burnette J.L., Pollack J.M., Forsyth R.B., Hoyt C.L., Babij A.D., Thomas F.N., Coy A.E. (2020). A growth mindset intervention: Enhancing students’ entrepreneurial self-efficacy and career development. Entrep. Theory Pract..

[24] Dweck C.S., Yeager D.S. (2019). Mindsets: A view from two eras. Perspect. Psychol. Sci..

[25] Lou N.M., Noels K.A. (2020). Breaking the vicious cycle of language anxiety: Growth language mindsets improve lower-competence ESL students’ intercultural interactions. Contemp. Educ. Psychol..

[26] Tweed R.G., Lehman D.R. (2002). Learning considered within a cultural context: Confucian and Socratic approaches. Am. Psychol..

[27] Wang J., Rao N. (2019). Classroom goal structures: Observations from urban and rural high school classes in China. Psychol. Sch..

[28] Veiga F., Leite A. (2016). Adolescents’ self-concept short scale: A version of PHCSCS. Procedia Soc..

[29] Trautwein U., Möller J. (2016). Self-Concept: Determinants and consequences of academic Self-Concept in school contexts. Plenum Series on Human Exceptionality.

[30] Lake J. (2015). Positive Psychology and Second Language Motivation: Empirically Validating A Model Of Positive L2 Self. Doctoral Dissertation.

[31] Byram M. (1997). Teaching and Assessing Intercultural Communicative Competence.

[32] Lapkin S., Swain M., Smith M. (2002). Reformulation and the learning of French pronominal verbs in a Canadian French immersion context. Mod. Lang. J..

[33] Yashima T. (2002). Willingness to communicate in a second language: The Japanese EFL context. Mod. Lang. J..

[34] Yashima T., Zenuk-Nishide L., Shimizu K. (2004). The influence of attitudes and affect on willingness to communicate and second language communication. Lang. Learn..

[35] Yu L. (2001). Communicative language teaching in China: Progress and resistance. TESOL Q..

[36] Early M., Norton B. (2014). Revisiting English as medium of instruction in rural African classrooms. J. Multiling. Multicult. Dev..

[37] Khajavy G.H., MacIntyre P.D., Hariri J. (2021). A closer look at grit and language mindset as predictors of foreign language achievement. Stud. Second Lang. Acquis..

[38] Wang H. (2023). The Roles of Classroom Social Climate, Language Mindset, and Positive L2 Self in Predicting Chinese College Students’ Academic Resilience in English as a Foreign Language. Unpublished Dissertation.

[39] UNESCO Education for Sustainability: From Rio to Johannesburg, Lessons Learnt from a Decade of Commitment. https://unesdoc.unesco.org/ark:/48223/pf0000127100.

[40] Rowe D. (2007). Education for a sustainable future. Science.

[41] Sterling S. (2001). Sustainable Education–Re-Visioning Learning and Change, Schumacher Briefing no 6.

[42] Sterling S., Gray D., Colucci-Gray L., Camino E. (2009). Sustainable Education. Science, Society and Sustainability: Education and Empowerment for an Uncertain World.

[43] Ben-Eliyahu A. (2021). Sustainable learning in education. Sustainability.

[44] Bronfenbrenner U. (1979). The Ecology of Human Development: Experiments by Nature and Design.

[45] Rogoff B. (2003). The Cultural Nature of Human Development.

[46] Beach K.D. (1999). Consequential transitions: A sociocultural expedition beyond transfer in education. Rev. Educ. Res..

[47] Hidi S.E., Renninger K.A. (2020). On educating, curiosity, and interest development. Curr. Opin. Behav. Sci..

[48] Biwer F., oude Egbrink M.G., Aalten P., de Bruin A.B. (2020). Fostering effective learning strategies in higher education—A mixed-methods study. J. Appl. Res. Mem. Cogn..

[49] Clifton R.A., Hamm J.M., Parker P.C. (2015). Promoting effective teaching and learning in higher education. High. Educ. Handb. Theory Res..

[50] Hativa N. (2001). Teaching for Effective Learning in Higher Education.

[51] Kinchin I.M., Lygo-Baker S., Hay D.B. (2008). Universities as centres of non-learning. High. Educ. Stud..

[52] Duckworth A.L., Peterson C., Matthews M.D., Kelly D.R. (2007). Grit: Perseverance and passion for long-term goals. J. Personal. Soc. Psychol..

[53] Boud D. (2007). Reframing assessment as if learning is important. Rethinking Assessment in Higher Education.

[54] Deardorff D.K. (2006). Identification and assessment of intercultural competence as a student outcome of internationalization. J. Stud. Int. Educ..

[55] Bagwe K.T., Haskollar E. (2020). Variables impacting intercultural competence: A systematic literature review. J. Intercult. Commun. Res..

[56] Borghetti C. (2013). Integrating intercultural and communicative objectives in the foreign language class: A proposal for the integration of two models. Lang. Learn. J..

[57] Hymes D., Pride J.B., Holmes J. (1972). On communicative competence. Sociolinguistics.

[58] Gao Y.H. (1998). The ‘Dao’ and ‘Qi’ concept of intercultural competence. Lang. Teach. Res..

[59] Jia Y.X. (2002). Intercultural Communication.

[60] Yang Y., Zhuang E.P. (2007). Framework for building cross-cultural communicative competence. Foreign Lang. Wor..

[61] Zhao A.G., Jiang Y.M. (2003). Introduction to Applied Language and Cultural Studies.

[62] Wu W.P., Fan W.W., Peng R.Z. (2013). An analysis of the assessment tools for Chinese college students’ intercultural competence. Foreign Lang. Teach. Res..

[63] Gu X. (2016). Assessment of intercultural communicative competence in FL education: A survey on EFL teachers’ perception and practice in China. Lang. Intercult. Commun..

[64] Huang Y. (2014). Constructing intercultural communicative competence framework for English learners. Cross-Cult. Comm..

[65] Ahnagari S., Zamanian J. (2014). Intercultural communicative competence in foreign language classroom. Int. J. Acad. Res. Bus. Soc. Sci..

[66] Fantini A.E. (2020). Reconceptualizing intercultural communicative competence: A multinational perspective. Res. Comp. Int. Educ..

[67] Fathi J., Pawlak M., Mehraein S., Hosseini H.M., Derakhshesh A. (2023). Foreign language enjoyment, ideal L2 self, and intercultural communicative competence as predictors of willingness to communicate among EFL learners. System.

[68] Mirzaei A., Forouzandeh F. (2013). Relationship between intercultural communicative competence and L2-learning motivation of Iranian EFL learners. J. Intercult. Commun. Res..

[69] Tran T.Q., Duong T.M. (2018). The effectiveness of the intercultural language communicative teaching model for EFL learners. Asian Pac. J. Sec. For..

[70] Badrkoohi A. (2018). The relationship between demotivation and intercultural communicative competence. Cogent Educ..

[71] Kanat-Mutluoğlu A. (2016). The influence of ideal L2 self, academic self-concept and intercultural communicative competence on willingness to communicate in a foreign language. Eurasian J. Appl. Linguist..

[72] Lou N.M., Noels K.A. (2016). Changing language mindsets: Implications for goal orientations and responses to failure in and outside the second language classroom. Contemp. Educ. Psychol..

[73] Lou N.M., Noels K.A. (2017). Measuring Language Mindsets and Modeling Their Relations with Goal Orientations and Emotional and Behavioral Responses in Failure Situations. Mod. Lang. J..

[74] Heyder A., Weidinger A.F., Steinmayr R. (2021). Only a burden for females in math? Gender and domain differences in the relation between adolescents’ fixed mindsets and motivation. J. Youth Adolesc..

[75] Peterman C.J., Ewing J. (2019). Effects of movement, growth mindset and math talks on math anxiety. J. Multi. Aff..

[76] Dörnyei Z. (2009). The L2 motivational self system. Motivation Lang. Identity L2 Self.

[77] Al-Hoorie A.H. (2018). The L2 motivational self system: A meta-analysis. Stud. Second Lang. Learn. Teach..

[78] Li M., Zhang L. (2021). Tibetan CSL learners’ L2 Motivational Self System and L2 achievement. System.

[79] Ebn-Abbasi F., Fattahi N., Noughabi M.A., Botes E. (2024). The strength of self and L2 willingness to communicate: The role of L2 grit, ideal L2 self and language mindset. System.

[80] Sadoughi M., Hejazi S.Y., Lou N.M. (2023). How do growth mindsets contribute to academic engagement in L2 classes? The mediating and moderating roles of the L2 motivational self system. Soc. Soc. Psychol. Educ..

[81] Eren A., Rakıcıoğlu-Söylemez A. (2020). Language mindsets, perceived instrumentality, engagement and graded performance in English as a foreign language students. Lang. Teach. Res..

[82] Wang H., Peng A., Patterson M.M. (2021). The roles of class social climate, language mindset, and emotions in predicting willingness to communicate in a foreign language. System.

[83] Peng R.Z., Wu W.P. (2016). Measuring intercultural contact and its effects on intercultural competence: A structural equation modeling approach. Int. J. Intercul. Rel..

[84] Peng R.Z., Wu W.P., Fan W.W. (2015). A comprehensive evaluation of Chinese college students’ intercultural competence. Int. J. Intercul. Rel..

[85] Peng R.Z., Zhu C., Wu W.P. (2020). Visualizing the knowledge domain of intercultural competence research: A bibliometric analysis. Int. J. Intercul. Rel..

[86] Rucker D.D., Preacher K.J., Tormala Z.L., Petty R.E. (2011). Mediation analysis in social psychology: Current practices and new recommendations. Soc. Personal. Psychol..

[87] Cheung G.W., Lau R.S. (2007). Testing mediation and suppression effects of latent variables: Bootstrapping with structural equation modeling. Organ. Res. Methods.

[88] Anderson J.C., Gerbing D.W. (1988). Structural equation modeling in practice: A review and recommended two-step approach. Psychol. Bull..

[89] Kline R.B. (2023). Principles and Practice of Structural Equation Modeling.

[90] Muthén L.K., Muthén B.O. (2012). Mplus User’s Guide (1998–2012).

[91] Bandalos D.L. (2002). The effects of item parceling on goodness-of-fit and parameter estimate bias in structural equation modeling. Struct. Equ. Model..

[92] Little T.D., Cunningham W.A., Shahar G., Widaman K.F. (2002). To parcel or not to parcel: Exploring the question, weighing the merits. Struct. Equ. Model..

[93] Marsh H.W., Hau K.T., Wen Z. (2004). In search of golden rules: Comment on hypothesis-testing approaches to setting cutoff values for fit indexes and dangers in overgeneralizing Hu and Bentler’s (1999) findings. Struct. Equ. Model..

[94] Schreiber J.B., Nora A., Stage F.K., Barlow E.A., King J. (2006). Reporting structural equation modeling and confirmatory factor analysis results: A review. J. Educ. Res..

[95] Valente M.J., Gonzalez O., Miocevic M., MacKinnon D.P. (2016). A note on testing mediated effects in Structural Equation Models: Reconciling past and current research on the performance of the test of joint significance. Educ. Psychol. Meas..

[96] Jin X. (2024). The role of effort in understanding academic achievements: Empirical evidence from China. Eur. J. Psychol. Educ..

[97] Young T.J., Sercombe P.G., Sachdev I., Naeb R., Schartner A. (2013). Success factors for international postgraduate students’ adjustment: Exploring the roles of intercultural competence, language proficiency, social contact and social support. Eur. J. High. Educ..

